# Associations between experience of stressful life events and cancer prevalence in China: results from the China Kadoorie Biobank study

**DOI:** 10.1186/s12885-023-11659-8

**Published:** 2023-11-24

**Authors:** Meng Wang, Weiwei Gong, Dianjianyi Sun, Pei Pei, Jun Lv, Canqing Yu, Min Yu

**Affiliations:** 1 Department of NCDs Control and Prevention, Zhejiang Provincial Center for Disease Control and Prevention, 3399 Binsheng Road, Hangzhou, 310051 China; 2 Department of Epidemiology & Biostatistics, School of Public Health, Peking University, Xueyuan Road, Haidian District, Beijing, 100191 China; 3 Peking University Center for Public Health and Epidemic Preparedness and Response, Xueyuan Road, Haidian District, Beijing, 100191 China

**Keywords:** Stressful life events, Cancer, Prevalence, Chinese

## Abstract

**Background:**

Studies examining the relationships of stressful life events and cancer yielded inconsistent findings, while relevant evidence in mainland China is scarce. The current study sought to determine whether experience of stressful life events was associated with cancer prevalence in Chinese population.

**Methods:**

We used cross-sectional data from the China Kadoorie Biobank study which that recruited 0.5 million Chinese adults aged 30 to 79 from 2004 to 2008. Logistic regression models were used to estimate adjusted odds ratios (ORs) with 95% confidence intervals (CIs) for cancer associated with stressful life events reported at baseline.

**Results:**

Among the 461,696 participants included in this analysis, 2,122 (0.46%) had self-reported cancer with the mean (SD) age was 57.12 (9.71) years. Compared to those without any stressful life event, participants who experienced 1 and 2 or more events had significantly higher odds of cancer, with the ORs of 1.80 (95% CI: 1.58–2.05) and 3.05 (2.18–4.28). For categories of work-, family-, and personal-related events, the OR of cancer was 1.48 (1.07–2.05), 2.06 (1.80–2.35), and 1.65 (1.17–2.33), respectively. Regarding the specific stressful life events, loss of income/living on debt, major conflict within family, death/major illness of other close family member, and major injury/traffic accident were significantly associated with increased odds of cancer, with the ORs of 2.64 (1.81–3.86), 1.73 (1.20–2.50), 2.36 (2.05–2.72), and 2.11 (1.43–3.13).

**Conclusion:**

Our findings suggested that experiences of cumulative and specific stressful life events were significantly associated with increased cancer prevalence in Chinese population.

**Supplementary Information:**

The online version contains supplementary material available at 10.1186/s12885-023-11659-8.

## Introduction

Cancer has become one of the most significant public health challenges of the 21st century. According to the GLOBOCAN 2020 estimated by International Agency for Research on Cancer (IRAC), approximately 19.3 million new cancer cases occurred worldwide and this figure is projected to reach 28.4 million in 2040 [1]. Cancer is also a major public health problem in China. It is estimated that nearly 4.6 million new cases have been diagnosed among Chinese population in 2020, accounting for the greatest global proportion (24% of the total) [2]. To better improve the current situation of cancer prevention and control, it is of great implications to understand more risk factors contributing to the cancer development. Although the exact etiology of cancer remains elusive, epidemiological evidence has proposed various modifiable lifestyle behaviors and environmental exposures related to cancer, including tobacco use, alcohol drinking, as well as physical inactivity, obesity, unhealthy diet, ultraviolet radiation, and air pollution [3].

In addition to these traditional risk factors, several stress-related psychological factors, such as depression, anxiety, stress prone personality and work stress have also been implicated in the pathogenesis of cancer [4–6]. Recently, experimental studies further indicated that psychological stress could influence the cancer initiation process through DNA damage, reactivation of oncogenic viruses and tumorigenesis [7, 8]. However, in the literature, available epidemiological evidence on the effect of psychological stress linked to cancer risk is not yet well-established, and stressful life events is of no exception. Specifically, findings from a recent systematic review and meta-analysis of cohort studies suggested that history of stressful life events was significantly associated with a moderate increase in the risk of breast cancer [9]. In a matched case-control study, Jafri, et al. found that lung cancer patients were more likely to have a major stressful life event in the past 5 years [10]. Besides, several studies further observed significant associations of certain stressful life events, such as personal illness and family members (e.g. child, parents, spouse) loss, with total and/or site-specific cancer risk [11–13]. Nevertheless, some other research on this topic yielded inconsistent findings. For example, a meta-analysis of 165 studies demonstrated that stress-related psychological factors were longitudinally associated with higher cancer incidence, which was not confirmed in the subgroup defined by stressful life experiences [14]. Similarly, results derived from a population-based birth cohort indicated that child loss was not associated with the parental risk of cancer disease [15]. In light of these inconsistent findings, more updated evidence is needed to address the possible linkage of stressful life events to cancer.

Notably, most existing evidence on these relationships came from Western studies, while little is known in China, where populations’ characteristics are significantly different from those in Western population. Therefore, with available baseline data from the China Kadoorie Biobank (CKB) study, we aimed to examine the associations between experience of stressful life events and cancer prevalence in the mainland China.

## Methods

### Study design and participants

Details about the CKB study design and participants have been described elsewhere [16–18]. Briefly, the baseline survey was conducted from 2004 to 2008 in 10 diverse regions (5 urban and 5 rural) in China, with 512,891 Chinese adults aged 30–79 years were finally recruited. Participants’ sociodemographic characteristics, smoking, alcohol drinking, diet, physical activity, general health (e.g., disease history and current medication use), family history of disease (e.g., diabetes and cancers), and reproductive history (only for women) were collected using an interviewer administered laptop-based questionnaire. Physical measurements (e.g., body weight, standing height, waist circumference, and blood pressure) were undertaken by trained health workers. The details of these factors could be found in the supplementary baseline questionnaire (S1).

### Assessment of outcome and exposure

Self-reported cancer was the outcome of interest in the present analysis. On the baseline survey, participants were asked whether a doctor had ever told them that they had the cancer with the dichotomized response options of “No” or “Yes”. If the answer was “Yes”, then the following site of cancer was further indicated ( lung, esophagus, stomach, liver, intestine, breast, prostate, cervix, and others. If there are more than one sites, choose the one that occurred first). Due to the relatively few cases of the specific site of cancer among participants, primary outcome for the main analysis in current study was total cancer.

The main exposure variable was stressful life events. Participants were asked to answer the question “Over the past 2 years have you had any of the following major events in your life?”, with the dichotomized response options “No” or “Yes” for each 10-item. Based on the Cobb-Clark and Schurer classifications [19], the 10-item life events in this study were categorized as follows: (1) work-related events, including loss of job or retirement, business bankruptcy, loss of income or living on debt; and (2) family-related events, including major conflict within the family, death or major illness of spouse or other close family member; and (3) personal-related events, including marital separation or divorce, victim of violence, major injury or traffic accident, or major natural disaster. Besides, the total number of stressful life events experienced was categorized as 0, 1 and 2 or more in the analysis.

### Statistical analysis

Among 512,891 participants in the baseline survey, we excluded those with a history of diabetes (n = 30,300), coronary heart disease (n = 15,472), stroke/transient ischemic attacks (n = 8,884), or psychiatric disorders (1,906). After these exclusions, 461,696 participants were included in the present analysis.

Multivariable logistic regression models were used to estimate the odds ratios (ORs) with 95% confidence intervals (CIs) for the associations of accumulative and specific stressful life events with cancer. Adjustments for confounding factors were conducted in four sequential models. In model 1, only sex (males, females) and birth cohorts (1920-1930 s, 1940s, 1950-1970 s) were adjusted. Model 2 was further adjusted for socioeconomic status including region (rural, urban), marriage status (unmarried, married), education (no formal school, primary school, middle school, high school, college/university), and household income (< 10 k, 10–20 k [10,000–19,999], 20–35 k [20,000–34,999], ≥ 35 k yuan). Model 3 was adjusted for all variables in model 2 plus health behaviors of smoking (never, occasional, current regular), alcohol drinking (never, occasional, current regular), physical activity (Metabolic Equivalents of Task, h/d), and anthropometric measurements including body mass index (BMI, kg/m^2^, underweight [<18.5], normal weight [18.5–23.9], overweight [24.0−27.9], obesity [≥ 28.0]) [20], waist circumference (with cut-off point of 90cm in males and 80cm in females). Model 4 was additionally adjusted for health status of hypertension, and family history of cancer. On the basis of model 4, associations of accumulative and specific stressful life events with cancer were compared within subgroups of participants defined by sex, region, birth cohorts, education, smoking, alcohol drinking, BMI, and hypertension. To evaluate the robustness of our estimates, sensitivity analysis was conducted with excluding those who smoked, drank alcohol, or had family history of cancer. Besides, we additionally explored the associations of accumulative and specific categories of stressful life events with specific sites of cancer in the same model. All analyses were performed using SAS version 9.4 (SAS Institute, Inc., Cary, NC) and R version 4.1.1 (The R Foundation for Statistical Computing). All statistical tests were based on the two-sided 5% level of significance.

## Results

### Characteristics of study participants

Among 461,696 participants included, the mean (SD) age was 51.23 (10.50) years. 41.04% of the participants were males, 57.73% were urban residents, and 18.45% had no formal education. Few participants were unmarried (8.90%) or obese (9.73%). At baseline, 2,122 (0.46%) participants had self-reported cancer with the mean (SD) age was 57.12 (9.71) years. Compared to those without any stressful life event, participants who had stressful life events tended to be younger, females, unmarried, less educated, and more physically active, with lower waist circumstance and lower proportion of smoking, alcohol drinking, and overweight/obesity (Table 1).

**Table 1:** Major baseline characteristics of CKB participants according to the number of stressful life events experienced in the past 2 years

Characteristics	Overall	No. of stressful life events		
		0	1	≥ 2
No. of participants	461,696	423,379	35,468	2,849
Mean age at baseline, year	51.23	51.25	51.05	49.98
Males, %	41.04	41.23	39.12	36.19
Birth cohorts, %				
1920-1930 s	10.18	10.25	9.68	7.23
1940s	19.85	19.85	20.00	18.29
1950-1970 s	69.96	69.90	70.32	74.48
Unmarried, %	8.90	7.97	18.18	31.17
Urban resident, %	57.73	58.22	52.14	53.14
No formal school, %	18.45	18.42	18.71	20.78
Lifestyle factors and anthropometric measurements, % or mean				
Current regular smoker	25.58	25.63	25.08	24.64
Current regular drinker	19.19	19.30	18.12	16.32
Body mass index, kg/m^2^				
Overweight (24.0-27.9)	32.33	32.42	31.50	28.92
Obesity (≥ 28.0 )	9.73	9.77	9.27	8.60
Waist circumference, cm	79.74	79.79	79.19	78.32
Physical activity, MET, h/d	21.83	21.81	22.00	22.56

CKB: China Kadoorie Biobank, MET: Metabolic Equivalents of Task

### Associations of accumulative and specific stressful life events with cancer

**Fig. 1:**
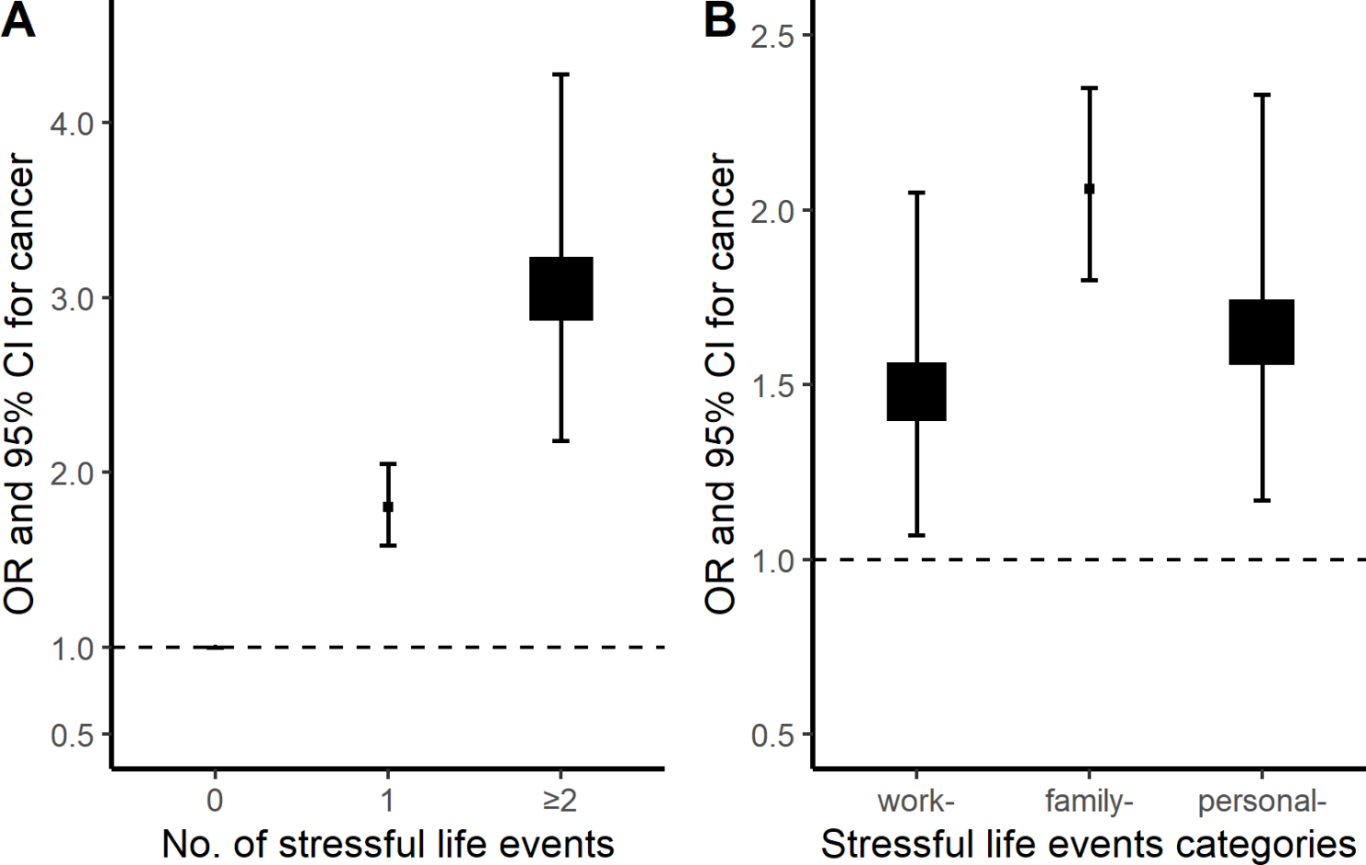
Associations of stressful life events with cancer. Specifically, (**A**) for accumulated number of stressful life events; (**B**) for categories of work-, family-, and personal-related stressful life events. Squares represent the adjusted odds ratios (ORs) with area inversely proportional to the number of cases. Vertical lines indicate the corresponding 95% confidence intervals (CIs)

After adjustment for potential confounders, including sociodemographic characteristics, lifestyle factors, and anthropometric measurements, compared to those without any stressful life event, participants who experienced 1 and 2 or more events had significantly higher odds of cancer, with the ORs of 1.80 (95% CI: 1.58–2.05) and 3.05 (2.18–4.28) (Table 2; Fig. 1A). For categories of work-, family-, and personal-related events, the OR of cancer was 1.48 (1.07–2.05), 2.06 (1.80–2.35), and 1.65 (1.17–2.33), respectively (Table 2; Fig. 1B). Regarding the specific stressful life events, loss of income/living on debt, major conflict within family, death/major illness of other close family member, and major injury/traffic accident were significantly associated with increased odds of cancer, with the ORs of 2.64 (1.81–3.86), 1.73 (1.20–2.50), 2.36 (2.05–2.72), and 2.11 (1.43–3.13) (Table 2). No heterogeneity was observed for the associations of accumulative and categories of stressful life events with cancer by sex, education, BMI, and hypertension (all P for heterogeneity ≥ 0.05), although some of these associations differed statistically as regards region, birth cohorts, smoking, and alcohol drinking (Supplementary Table 1). In the sensitivity analysis, although the ORs varied slightly, the associations of accumulative and specific stressful life events with cancer were broadly consistent among the subset of participants without smoking, drinking alcohol, or family history of cancer (Supplementary Table 2). Additionally, these associations for some sites of cancer were consistent with results in the main analyses (Supplementary Table 3).

**Table 2:** Adjusted odds ratios (95% CIs) of cancer by the stressful life events experienced in the past 2 years

Stressful life events	Cases/total	Model 1	Model 2	Model 3	Model 4
No. of stressful life events (vs. 0)					
1	290/35,468	1.94 (1.71–2.20)*	1.91 (1.68–2.17)*	1.95 (1.72–2.21)*	1.80 (1.58–2.05)*
≥ 2	37/2,849	3.24 (2.33–4.50)*	3.25 (2.34–4.53)*	3.18 (2.28–4.44)*	3.05 (2.18–4.28)*
Work-related events (yes vs. no)	40/5,367	1.90 (1.39–2.60)*	1.76 (1.29–2.42)*	1.50 (1.09–2.06)*	1.48 (1.07–2.05)*
Loss of job/retirement	12/2,042	1.64 (0.93–2.89)	1.33 (0.75–2.35)	0.94 (0.53–1.68)	0.90 (0.50–1.64)
Business bankruptcy	5/1,093	1.15 (0.48–2.76)	1.19 (0.49–2.86)	1.28 (0.53–3.09)	1.24 (0.51–2.30)
Loss of income/living on debt	29/2,420	2.88 (1.99–4.17)*	2.82 (1.95–4.09)*	2.59 (1.79–3.77)*	2.64 (1.81–3.86)*
Family-related events (yes vs. no)	281/29,737	2.15 (1.90–2.44)*	2.14 (1.88–2.43)*	2.22 (1.95–2.52)*	2.06 (1.80–2.35)*
Major conflict within family	31/3,989	1.70 (1.19–2.43)*	1.67 (1.17–2.39)*	1.76 (1.23–2.52)*	1.73 (1.20–2.50)*
Death/major illness of spouse	17/3,932	0.69 (0.43–1.11)	0.66 (0.40–1.08)	0.66 (0.40–1.07)	0.61 (0.37–1.02)
Death/major illness of other close family member	238/22,580	2.51 (2.19–2.88)*	2.46 (2.15–2.82)*	2.57 (2.24–2.95)*	2.36 (2.05–2.72)*
Personal-related events (yes vs. no)	36/5,213	1.72 (1.23–2.39)*	1.69 (1.22–2.36)*	1.80 (1.29–2.51)*	1.65 (1.17–2.33)*
Marital separation/divorce	4/1,226	0.93 (0.35–2.48)	0.80 (0.30–2.14)	0.87 (0.32–2.33)	0.66 (0.21–2.08)
Victim of violence	2/638	0.76 (0.19–3.03)	0.76 (0.19–3.06)	0.79 (0.20–3.18)	0.77 (0.19–3.10)
Major injury/traffic accident	27/2,979	2.16 (1.47–3.16)*	2.21 (1.51–3.23)*	2.29 (1.56–3.36)*	2.11 (1.43–3.13)*
Major natural disaster	3/446	1.62 (0.52–5.04)	1.69 (0.54–5.27)	2.08 (0.67–6.51)	2.02 (0.65–6.32)

Model 1 adjusted for sex and birth cohort; model 2 adjusted for model 1 plus socioeconomic status including area, marriage status, education, and household income; model 3 adjusted for model 2 plus health behaviors of smoking, alcohol drinking, physical activity (Metabolic Equivalents of Task, h/d), and anthropometric measurements including body mass index, waist circumference; model 4 adjusted for model 3 plus health status of hypertension, and family history of cancer

* Significant results

## Discussion

To our knowledge, this is the first large epidemiological study to examine whether experience of stressful life events was associated with cancer prevalence in China. Based on nearly 500,000 adults aged 30–79 years from 10 diverse regions in China, we found that participants with cumulative stressful life events in the 2 years preceding the survey had significantly higher odds of cancer, after adjustment for potential confounders. Specific to categories of work-, family-, and personal-related stressful life events, similar positive associations with cancer prevalence were also observed in this study. All these reported relationships were broadly consistent across most subgroups and in the sensitivity analysis after excluding participants with smoking, alcohol drinking and family history of cancer. Moreover, although the cases of specific cancer sites were relatively limited, some consistent linkages with the stressful life events found simultaneously reinforced our main findings.

Since the differences in the measure of stressful life events, study design, characteristic of participants, and potential confounder adjustment across the literature, our study joined the previous evidence showing a relationship between cumulative stressful life events and higher odds of cancer, including the breast cancer that is mostly discussed at present. For example, case-control study separately conducted in USA and Israel showed that as the number of stressful life events increased, the odds of breast cancer among women increased as well [12, 21]. Similarly, findings from the Finnish Twin Cohort study also suggested a significant association between the accumulation of major life events during 5 years prior to baseline survey and an increased risk of breast cancer during 15 years of follow-up [22]. However, currently, these associations are still under dispute with the example of another prospective cohort study conducted in Australasian families reporting that the total number of acute or chronic stressful events was not associated with breast cancer risk [23]. Besides, regarding the cancer-related effects of cumulative stressful life, given most prior research focused on the breast site, more prospective studies involved in the total and other specific sites cancer are warranted.

In the present study, we further examined the associations between certain stressful life events and cancer individually. According to the analyses, relevant results suggested that the identified categories of work-, family-, and personal-related events were consistently associated with increased odds of cancer. Similarly, positive linkages to cancer were also observed in the specific life events of loss of income/living on debt, major conflict within family, death/major illness of other close family member, and major injury/traffic accident. Overall, the higher odds of cancer we found in participants experiencing these certain life events is relatively consistent with observations in the literature. For example, similar to our study on loss of income/living on debt, several previous studies have confirmed that financial difficulties or poverty played an important role in morbidity caused by breast, prostate and colorectal cancer [24–26]. Besides, findings from case-control studies conducted in Iran and Serbia consistently showed that death of close family members was associated with the increased odds of colorectal and breast cancer [27, 28]. Another case-control study from Poland also suggested that major life event of personal injury or illness was implicated in the etiology of breast cancer [29]. Meanwhile, we reported a significant association between major conflict within family and higher odds of cancer, and however, this did not remain statistical significant with life event of family and husband disputes in another study [27]. Interestingly, our findings also showed that death/major illness of spouse was marginally associated with the decreased cancer prevalence, which was rarely reported by the previous studies. In the Holmes-Rahe Social Readjustment Rating Scale [30], death of a spouse and divorce received the highest scores, with death of a close family member being rated fifth. Therefore, given the positive association of death/major illness of other close family member with increased cancer prevalence in this study, our reports of the marginally protective association with death/major illness of spouse might be a chance finding.

Possible biological mechanisms accounting for the stress-cancer linkage have been studied extensively and one hypothesized mechanism is that dysregulation of the hypothalamus-pituitary-adrenal axis caused by stressful life events may result in increased cortisol through activation of the sympathetic nervous system. These cause physiological and metabolic changes including influencing the inflammatory response and suppressing the immune system [31–33].

There are several strengths of the present study, including the large sample size and diversity of areas covered, contributing to the generalization of study findings to general Chinese population. Moreover, the completeness of data collection and the wide adjustment for potential confounders simultaneously limit the possible confounding bias in the analyses. However, the study also has some limitations. First, despite a relatively comprehensive set of potential confounders has been considered in analysis, residual confounding from unmeasured factors cannot be ruled out. Second, based on a cross-sectional design, the present study is not capable of identifying the causality of reported associations between stressful life events and cancer in Chinese population. Third, the number of self-reported cancer cases and some certain stressful life events is relatively limited, which may decrease the statistical power to examine their associations and affect the reliability of observations in this study. Fourth, the baseline information of cancer assessment is relied on self-reports and may have been subject to reporting bias, resulting in misclassification of the disease status.

## Conclusions

In conclusion, results of our large epidemiological study supported that cumulative and specific stressful life events were significantly associated with increased odds of cancer in China. Based on these findings, we recommended that relevant approaches evaluating the cancer risk among population should consider the individual experience of stressful life events seriously.

### Electronic supplementary material

Below is the link to the electronic supplementary material.

Supplementary Material 1

Supplementary Material 2

## Data Availability

The datasets used and/or analysed during the current study are available from the corresponding author on reasonable request.

## Abbreviations

IRAC: International Agency for Research on Cancer
CKB: China Kadoorie Biobank
OR: Odds ratio
CI: Confidence interval
BMI: Body mass index
