# Supplementary material for: Associations between experience of stressful life events and cancer prevalence in China: results from the China Kadoorie Biobank study
Source: BMC Cancer. 2023 Nov 24;23:1142. doi: 10.1186/s12885-023-11659-8 (PMC10675951; doi:10.1186/s12885-023-11659-8)
Supplement: Supplementary file 1 — Supplementary Material 1 [file 12885_2023_11659_MOESM1_ESM.docx]

| Supplementary Table 1. Adjusted odds ratios (95% CIs) of cancer by the stressful life events experienced in the past 2 years within different subgroups. | | | | | | | |
| --- | --- | --- | --- | --- | --- | --- | --- |
| Characteristics | Cases/total | No. of stressful life events (vs. 0) | |  | Specific stressful life events (vs. no) | | |
|  |  | 1 | ≥2 |  | Work-related | Family-related | Personal-related |
| Sex |  |  |  |  |  |  |  |
| Males | 806/189,480 | 2.11(1.71-2.59)* | 4.16(2.48-6.98)* |  | 1.95(1.23-3.09)* | 2.41(1.95-2.98)* | 2.01(1.21-3.33)* |
| Females | 1,316/272,216 | 1.62(1.37-1.92)* | 2.55(1.63-4.00)* |  | 1.18(0.74-1.89) | 1.86(1.57-2.20)* | 1.42(0.89-2.28) |
| Heterogeneity test: χ^2^ (P) |  | 3.75 (0.05) | 1.96 (0.16) |  | 2.24 (0.13) | 3.51 (0.06) | 0.97 (0.32) |
| Region |  |  |  |  |  |  |  |
| Rural | 1,035/266,515 | 1.89(1.56-2.29)* | 2.65(1.54-4.55)* |  | 1.23(0.66-2.31) | 2.22(1.83-2.69)* | 1.00(0.54-1.88) |
| Urban | 1,087/195,181 | 1.74(1.45-2.08)* | 3.36(2.18-5.18)* |  | 1.59(1.08-2.33)* | 1.94(1.62-2.33)* | 2.24(1.48-3.38)* |
| Heterogeneity test: χ^2^ (P) |  | 0.38 (0.54) | 0.45 (0.50) |  | 0.47 (0.49) | 1.00 (0.32) | 4.47 (0.04)* |
| Birth cohorts |  |  |  |  |  |  |  |
| 1920s-1930s | 429/47,022 | 1.11(0.77-1.60) | 1.15(0.28-4.68) |  | NA | 1.22(0.85-1.76) | 0.85(0.21-3.45) |
| 1940s | 650/91,660 | 1.86(1.47-2.34)* | 1.15(0.43-3.12) |  | 0.38(0.09-1.51) | 1.93(1.52-2.46)* | 1.71(0.88-3.33) |
| 1950s-1970s | 1,043/323,014 | 1.99(1.67-2.37)* | 4.25(2.92-6.19)* |  | 1.81(1.28-2.54)* | 2.48(2.07-2.96)* | 1.67(1.09-2.54)* |
| Heterogeneity test: χ^2^ (P) |  | 8.05 (0.02)* | 8.23 (0.02)* |  | NA | 12.34 (0.02)* | 0.86 (0.65) |
| Education |  |  |  |  |  |  |  |
| No formal school | 391/85,205 | 2.22(1.67-2.96)* | 2.92(1.36-6.28)* |  | 1.77(0.78-4.03) | 2.46(1.85-3.28)* | 1.25(0.51-3.06) |
| Primary school | 771/148,503 | 1.76(1.40-2.20)* | 2.78(1.55-5.00)* |  | 1.51(0.83-2.76) | 2.00(1.59-2.51)* | 1.55(0.87-2.76) |
| Middle school or higher | 960/227,988 | 1.70(1.39-2.06)* | 3.34(2.04-5.47)* |  | 1.47(0.95-2.29) | 1.95(1.60-2.38)* | 1.84(1.13-3.00)* |
| Heterogeneity test: χ^2^ (P) |  | 2.40 (0.30) | 0.24 (0.88) |  | 0.15 (0.93) | 1.83 (0.40) | 0.60 (0.74) |
| Regular smoking |  |  |  |  |  |  |  |
| No | 1,880/343,603 | 1.79(1.56-2.06)* | 2.70(1.84-3.95)* |  | 1.37(0.96-1.97) | 2.06(1.79-2.37)* | 1.33(0.89-2.00) |
| Yes | 242/118,093 | 1.84(1.26-2.69)* | 5.46(2.62-11.37)* |  | 2.08(0.96-4.51) | 2.01(1.36-2.96)* | 3.69(1.92-7.07)* |
| Heterogeneity test: χ^2^ (P) |  | 0.02 (0.89) | 2.78 (0.10) |  | 0.92 (0.34) | 0.01 (0.91) | 6.79 (0.01)* |
| Regular alcohol drinking |  |  |  |  |  |  |  |
| No | 1,923/373,098 | 1.88(1.64-2.15)* | 2.90(2.01-4.16)* |  | 1.48(1.05-2.09)* | 2.17(1.89-2.49)* | 1.34(0.90-2.00) |
| Yes | 199/88,598 | 1.20(0.72-1.98) | 4.90(1.95-12.31)* |  | 1.73(0.63-4.75) | 1.06(0.60-1.88) | 4.63(2.32-9.22)* |
| Heterogeneity test: χ^2^ (P) |  | 2.82 (0.09) | 1.08 (0.30) |  | 0.08 (0.78) | 5.71 (0.02)* | 9.29 (0.002)* |
| Body mass index, ㎏/㎡ |  |  |  |  |  |  |  |
| <18.5 | 199/20,917 | 1.71(1.10-2.65)* | 4.98(2.09-11.92)* |  | 1.42(0.44-4.57) | 2.07(1.35-3.17)* | 2.74(1.08-6.95)* |
| 18.5-23.9 | 1,061/246,603 | 1.87(1.56-2.24)* | 2.47(1.47-4.16)* |  | 1.33(0.82-2.17) | 2.16(1.79-2.59)* | 1.18(0.68-2.05) |
| 24.0-27.9 | 638/149,266 | 1.85(1.45-2.34)* | 3.24(1.71-6.12)* |  | 1.95(1.16-3.28)* | 2.00(1.56-2.57)* | 1.88(1.03-3.44)* |
| ≥28.0 | 224/44,908 | 1.39(0.89-2.18) | 3.89(1.56-9.73)* |  | 1.09(0.34-3.46) | 1.69(1.09-2.63)* | 2.69(1.09-6.62)* |
| Heterogeneity test: χ^2^ (P) |  | 1.55 (0.67) | 2.11 (0.55) |  | 1.50 (0.68) | 1.08 (0.78) | 3.78 (0.29) |
| Hypertension |  |  |  |  |  |  |  |
| No | 1,890/419,837 | 1.82(1.58-2.09)* | 3.23(2.28-4.57)* |  | 1.51(1.08-2.11)* | 2.08(1.81-2.40)* | 1.78(1.26-2.51)* |
| Yes | 232/41,859 | 1.63(1.08-2.46)* | 1.63(0.40-6.71) |  | 1.02(0.25-4.15) | 1.85(1.23-2.77)* | NA |
| Heterogeneity test: χ^2^ (P) |  | 0.25 (0.62) | 0.85 (0.36) |  | 0.28 (0.59) | 0.29 (0.59) | NA |
| All models were adjusted for sex, birth cohort, area, marriage status, education, household income, smoking, alcohol drinking, physical activity (Metabolic Equivalents of Task, h/d), body mass index, waist circumference, hypertension, and family history of cancer.  * Significant results. | | | | | | | |

| Supplementary Table 2. Sensitivity analysis: adjusted odds ratios (95% CIs) of cancer by the stressful life events experienced in the past 2 years only among participants without smoking, alcohol drinking and family history of cancer. | | | | | |
| --- | --- | --- | --- | --- | --- |
| Stressful life events | Cases/total | Model 1 | Model 2 | Model 3 | Model 4 |
| No. of stressful life events (vs. 0) |  |  |  |  |  |
| 1 | 125/10,784 | 2.56 (2.11-3.11)* | 2.60 (2.13-3.16)* | 2.64 (2.17-3.22)* | 2.65 (2.18-3.23)* |
| ≥2 | 15/924 | 3.79 (2.26-6.36)* | 3.89 (2.31-6.56)* | 3.87 (2.29-6.53)* | 3.88 (2.30-6.55)* |
| Work-related events (yes vs. no) | 14/1,630 | 1.87 (1.10-3.19)* | 1.75 (1.03-2.99)* | 1.57 (0.92-2.69) | 0.64 (0.37-1.09) |
| Loss of job/retirement | 3/589 | 1.09 (0.35-3.41) | 0.90 (0.29-2.82) | 0.71 (0.23-2.22) | 0.71 (0.23-2.22) |
| Business bankruptcy | 1/218 | 1.02 (0.14-7.31) | 1.04 (0.15-7.45) | 1.07 (0.15-7.63) | 1.06 (0.15-7.62) |
| Loss of income/living on debt | 10/876 | 2.49 (1.33-4.66)* | 2.48 (1.32-4.67)* | 2.40 (1.27-4.51)* | 2.39 (1.27-4.51)* |
| Family-related events (yes vs. no) | 124/1,630 | 2.90 (2.39-3.52)* | 2.96 (2.43-3.61)* | 3.05 (2.50-3.71)* | 3.06 (2.51-3.72)* |
| Major conflict within family | 11/1,197 | 1.93 (1.06-3.52)* | 1.90 (1.05-3.47)* | 1.98 (1.09-3.61)* | 1.99 (1.09-3.63)* |
| Death/major illness of spouse | 5/1,575 | 0.54 (0.22-1.31) | 0.50 (0.20-1.22) | 0.50 (0.20-1.24) | 0.50 (0.20-1.24) |
| Death/major illness of other close family member | 111/6,744 | 3.61 (2.95-4.42)* | 3.65 (2.98-4.48)* | 3.76 (3.07-4.62)* | 3.77 (3.08-4.63)* |
| Personal-related events (yes vs. no) | 14/1,455 | 2.02 (1.18-3.44)* | 1.99 (1.17-3.40)* | 2.16 (1.26-3.69)* | 2.16 (1.27-3.69)* |
| Marital separation/divorce | 0/282 | NA | NA | NA | NA |
| Victim of violence | 1/161 | 1.23 (0.17-8.84) | 1.23 (0.17-8.85) | 1.36 (0.19-9.76) | 1.37 (0.19-9.83) |
| Major injury/traffic accident | 11/892 | 2.53 (1.39-4.61)* | 2.57 (1.41-4.69)* | 2.73 (1.50-4.99)* | 2.73 (1.50-4.99)* |
| Major natural disaster | 2/144 | 2.96 (0.73-12.01) | 3.13 (0.77-12.73) | 3.94 (0.96-16.13) | 3.96 (0.97-16.21) |
| Model 1 adjusted for sex and birth cohort; model 2 adjusted for model 1 plus socioeconomic status including area, marriage status, education, and household income; model 3 adjusted for model 2 plus health behavior of physical activity (Metabolic Equivalents of Task, h/d), and anthropometric measurements including body mass index, waist circumference; model 4 adjusted for model 3 plus health status of hypertension.  * Significant results. | | | | | |

| Supplementary Table 3. Adjusted odds ratios (95% CIs) of specific site of cancer by the stressful life events experienced in the past 2 years. | | | | | | | |
| --- | --- | --- | --- | --- | --- | --- | --- |
| Site of cancer | No. of cases | No. of stressful life events (vs. 0) | |  | Specific categories of stressful life events (vs. no) | | |
|  |  | 1 | ≥2 |  | Work-related | Family-related | Personal-related |
| Lung | 102 | 2.08 (1.15-3.77)* | 4.58 (1.10-19.00)* |  | 2.47 (0.77-7.95) | 1.87 (0.96-3.63) | 2.29 (0.56-9.39) |
| Esophagus | 261 | 1.04 (0.66-1.64) | 0.57 (0.08-4.08) |  | 1.16 (0.37-3.65) | 1.03 (0.63-1.68) | 0.69 (0.17-2.78) |
| Stomach | 217 | 1.96 (1.34-2.88) | 2.41 (0.76-7.64) |  | 0.38 (0.05-2.71) | 2.48 (1.70-3.60)* | 1.33 (0.42-4.19) |
| Liver | 33 | 3.73 (1.64-8.49)* | 5.86 (0.77-44.85) |  | 1.39 (0.18-10.62) | 5.67 (2.59-12.44)* | NA |
| Intestine | 239 | 2.27 (1.58-3.27)* | 2.68 (0.85-8.48) |  | 1.64 (0.60-4.44) | 2.12 (1.43-3.13)* | 3.98 (1.95-8.11)* |
| Prostate | 5 | 4.52 (0.43-48.11) | NA |  | NA | 8.91 (1.29-61.52)* | 48.28 (4.40-529.74)* |
| Breast | 456 | 1.64 (1.23-2.17)* | 2.64 (1.24-5.62)* |  | 1.86 (1.01-3.41)* | 1.81 (1.36-2.42)* | 0.67 (0.21-2.09) |
| Cervix | 307 | 1.64 (1.15-2.35)* | 2.92 (1.19-7.14)* |  | 0.67 (0.17-2.69) | 1.92 (1.35-2.74)* | 1.78 (0.73-4.33) |
| All models were adjusted for sex, birth cohort, area, marriage status, education, household income, smoking, alcohol drinking, physical activity (Metabolic Equivalents of Task, h/d), body mass index, waist circumference, hypertension, and family history of cancer.  * Significant results. | | | | | | | |
